# Tumor Energy Metabolism and Potential of 3-Bromopyruvate as an Inhibitor of Aerobic Glycolysis: Implications in Tumor Treatment

**DOI:** 10.3390/cancers11030317

**Published:** 2019-03-06

**Authors:** Tengjiao Fan, Guohui Sun, Xiaodong Sun, Lijiao Zhao, Rugang Zhong, Yongzhen Peng

**Affiliations:** 1 Beijing Key Laboratory of Environmental & Viral Oncology, College of Life Science & Bioengineering, Beijing University of Technology, Beijing 100124, China; fantengjiao2014@emails.bjut.edu.cn (T.F.); sunxd@emails.bjut.edu.cn (X.S.); zhaolijiao@bjut.edu.cn (L.Z.); lifesci@bjut.edu.cn (R.Z.); 2 National Engineering Laboratory for Advanced Municipal Wastewater Treatment & Reuse Technology, Engineering Research Center of Beijing, Beijing University of Technology, Beijing 100124, China; pyz@bjut.edu.cn

**Keywords:** aerobic glycolysis, tumor energy metabolism, 3-bromopyruvate, glycolytic inhibitor, antitumor effect

## Abstract

Tumor formation and growth depend on various biological metabolism processes that are distinctly different with normal tissues. Abnormal energy metabolism is one of the typical characteristics of tumors. It has been proven that most tumor cells highly rely on aerobic glycolysis to obtain energy rather than mitochondrial oxidative phosphorylation (OXPHOS) even in the presence of oxygen, a phenomenon called “Warburg effect”. Thus, inhibition of aerobic glycolysis becomes an attractive strategy to specifically kill tumor cells, while normal cells remain unaffected. In recent years, a small molecule alkylating agent, 3-bromopyruvate (3-BrPA), being an effective glycolytic inhibitor, has shown great potential as a promising antitumor drug. Not only it targets glycolysis process, but also inhibits mitochondrial OXPHOS in tumor cells. Excellent antitumor effects of 3-BrPA were observed in cultured cells and tumor-bearing animal models. In this review, we described the energy metabolic pathways of tumor cells, mechanism of action and cellular targets of 3-BrPA, antitumor effects, and the underlying mechanism of 3-BrPA alone or in combination with other antitumor drugs (e.g., cisplatin, doxorubicin, daunorubicin, 5-fluorouracil, etc.) in vitro and in vivo. In addition, few human case studies of 3-BrPA were also involved. Finally, the novel chemotherapeutic strategies of 3-BrPA, including wafer, liposomal nanoparticle, aerosol, and conjugate formulations, were also discussed for future clinical application.

## 1. Introduction

Global cancer statistics 2018 released by the International Agency for Research on Cancer (IARC) estimates that there would be 18.1 million new cancer cases and 9.6 million cancer deaths in 2018, cancer is expected to rank as the leading cause of death [1]. This serious situation stimulates us to further reveal the pathogenesis of cancer, establish effective prevention methods, and search new screening and diagnostic methods and new treatment methods for cancer. As we know, as a consequence of numerous mutations, tumor cells exhibit various different biological metabolism processes with respect to normal cells [2].

**Figure 1:**
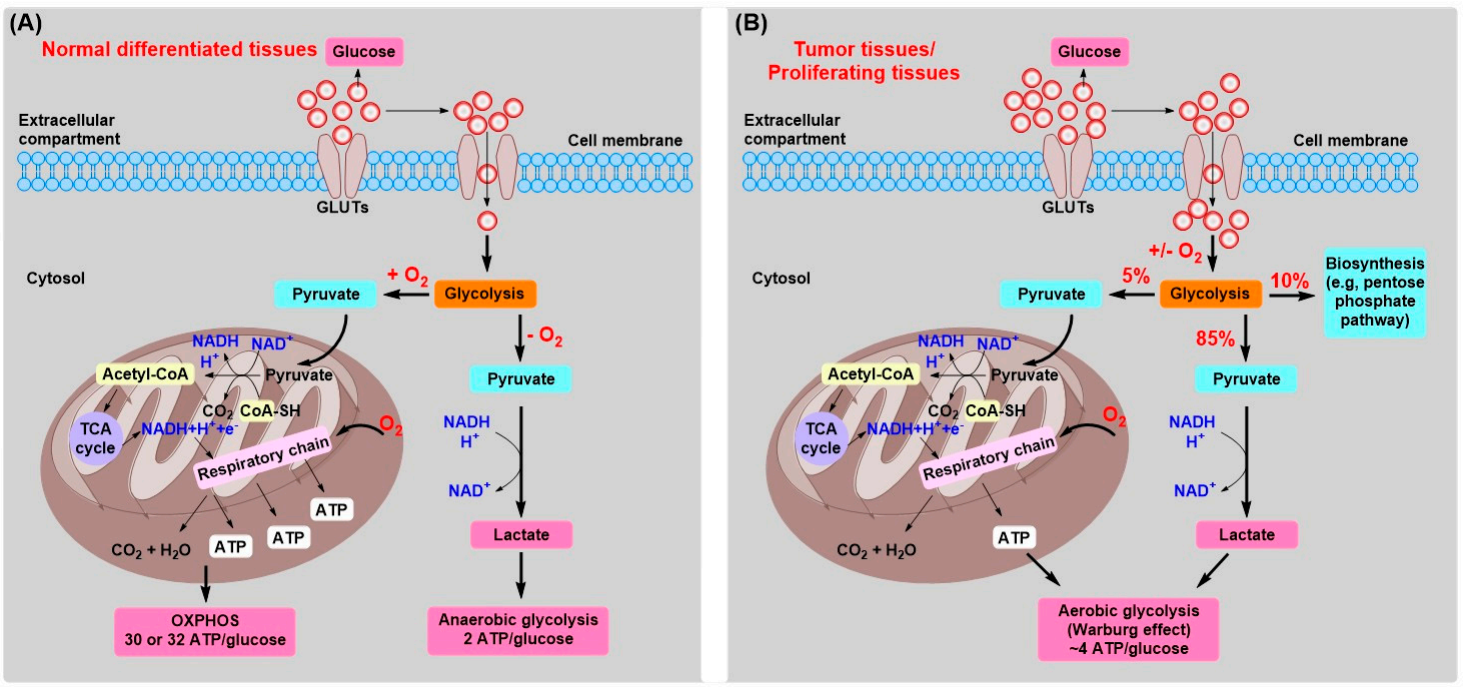
The energy metabolism of normal differentiated tissues and tumor or proliferating tissues. (**A**) In normal differentiated tissues, under aerobic condition, a molecule of glucose is first converted to two pyruvates via glycolysis in the cytosol, followed by undergoing TCA cycle to produce CO_2_ in the mitochondria. A total of 30 or 32 ATP molecules are generated during this process. Under anaerobic condition, glycolysis is preferential and less pyruvate is shifted to the oxygen-consuming mitochondria, only 2 ATP molecules are produced per molecule of glucose. (**B**) In tumor or proliferating tissues, mitochondrial function is still normal, but little mitochondrial oxidative phosphorylation (OXPHOS) continues in tumor cells. In order to satisfy the metabolic requirements of both energy and materials for rapidly proliferating cells, ~85% of the glucose is processed to lactate via glycolytic pyruvate even in the presence of oxygen and ~5% of the glucose is metabolized by OXPHOS. In addition, ~10% of the glucose is diverted into the upstream of pyruvate production for biosynthesis (e.g., pentose phosphate pathway, PPP).

The first found tumor-specific alteration in metabolism is the reprogrammed energy metabolism, which was first discovered by German biochemists Otto Warburg and coworkers in the 1920s [2,3,4]. Now this phenomenon is commonly termed “Warburg effect”, which is one of the typical hallmarks of cancers [2,5,6]: (1) normal cells produce energy (adenosine triphosphate, ATP) primarily via mitochondrial oxidative phosphorylation (OXPHOS), while most tumor cells tend to produce energy mainly through a high rate of glycolysis even in the presence of oxygen, a reprogrammed metabolism characterized by high glucose consumption, followed by elevated production of lactate; the “Warburg effect” is thus also called “aerobic glycolysis”. (2) Compared to mitochondrial OXPHOS, the “Warburg effect” is less efficient in terms of ATP production. Under aerobic condition, a molecule of glucose is first converted to two pyruvates via glycolysis in the cytosol, followed by undergoing tricarboxylic acid (TCA) cycle to produce CO_2_ in the mitochondria. A total of 30 or 32 ATP molecules are generated during this process (Figure 1A). However, under anaerobic condition, glycolysis is preferential and less pyruvate is shifted to the oxygen-consuming mitochondria. Only two ATP molecules are produced through glycolysis under anaerobic conditions per molecule of glucose (Figure 1A), with nearly 16-fold lower efficiency of energy production by glycolysis compared to OXPHOS.

Why tumor cells select an inefficient energy production for cellular processes? To date, although the “Warburg effect” has been widely studied, the precise advantage of glycolysis confers to tumors cells remains elusive. However, the crucial perspectives in terms of “Warburg effect”, characterized by most tumor cells, are described in the following section. Based on the current knowledge of “Warburg effect”, its inhibition may open a feasible window for cancer treatment.

## 2. Tumor Energy Metabolism: Target for Tumor Chemotherapy

Initially, Warburg postulated that the unique energy metabolism is derived from the damage, defect, or low content of mitochondria in tumor cells, then leading to impaired aerobic aspiration and a subsequent high dependence on aerobic glycolysis [4,7,8]. This alteration in energy metabolism is initially considered as the fundamental cause of cancer, a claim now known as the “Warburg hypothesis” [4,9]. However, the following studies indicated that most tumor cells have nonimpaired mitochondrial function, suggesting an additional existed mechanism responsible for “Warburg effect” in tumor cells [5]. Up to this day, mutations of oncogenes and tumor suppressor genes are regarded as the main cause of malignant transformation, and aerobic glycolysis is only an epiphenomenon that results from these complex mutations rather than a cause of cancer [2,10]. In fact, not only glycolysis, but also the TCA cycle, fatty acid β-oxidation, and anabolic metabolism are both reprogrammed to adapt to the new function of tumor cells [5,11,12].

At first glance, a possible question may come to our mind that “why a less efficient aerobic glycolysis is chosen by most tumor cells in terms of the energy production.” It should be noted that, to a great extent, our current knowledge regarding cellular metabolism process primarily relies on the studies of nonproliferating cells within differentiated tissues [5]. These cells mainly metabolize glucose to CO_2_ through the complete mitochondrial oxidation of pyruvate generated from glycolysis, and then a large amount of ATP molecules are produced. For proliferating cells, in addition to energy requirement, they must require a number of building blocks for macromolecular synthesis, including nucleotides, lipids, and amino acids. Interestingly, many unicellular organisms preferentially utilize “fermentation”—similar to aerobic glycolysis in tumor cells—to fuel cell growth and division even in the presence of oxygen. It also warrants the aerobic glycolysis can provide sufficient energy for cell proliferation.

**Figure 2:**
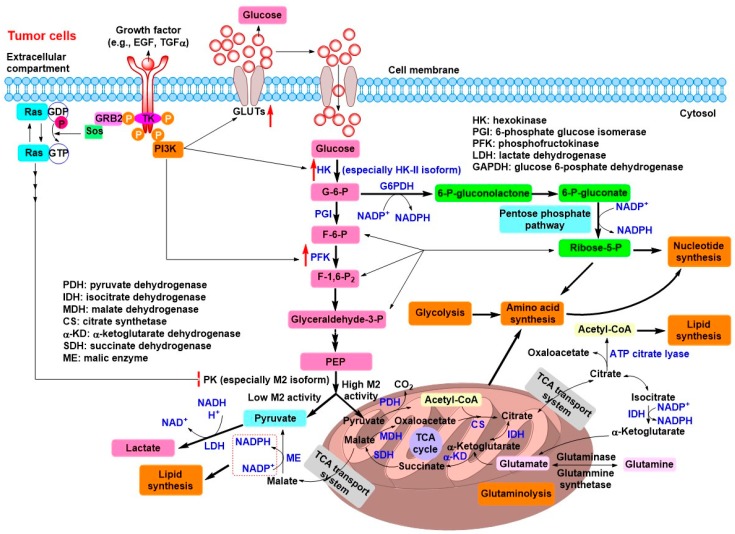
Schematic representation of metabolism in tumor cells. Mutation-mediated continuous activation of PI3K/AKT signaling pathway upregulates the expression of glucose transporters (GLUTs) and substantially enhances the capture of glucose into the cytoplasm by HK, and activates PFK or upregulates PFK expression, leading to the high rate of glucose influx, which in turn facilitates the aerobic glycolysis. The glycolytic switch in tumor cells allows the direct or indirect flux of glycolytic intermediates to many biosynthetic pathways (e.g., PPP, amino acid synthesis, lipid synthesis, and nucleotide synthesis), which provides the biomacromolecules and other materials required for producing new daughter cells. In addition, the intermediates of glutaminolysis are also used for synthesizing biomass that rapidly growing tumor cells need.

Based on these findings, currently, there are several rational explanations with respect to “Warburg effect” occurred in most tumor cells [5]. First, only when resources are scarce, inefficient aerobic glycolysis is a serious problem for cell proliferation. Secondly, the glycolytic switch in tumor cells allows the flux of glycolytic intermediates to many biosynthetic pathways, which in turn facilitates the synthesis of the biomacromolecules and other materials required for producing daughter cells (Figure 2). For proliferating tumor cells, to replicate all cellular components, they have a large need for nucleotides, lipids, and amino acids. If glucose is used to generate ATP through mitochondrial OXPHOS, the carbon sources for biomacromolecules synthesis would be severely limited. For example, the ATP requirement for synthesizing a 16-carbon fatty acid palmate, a major component in cell membranes, is five times less than the OXPHOS of a glucose molecule could provide, while seven glucose molecules must supply the NADPH required. In addition, at least three glucose molecules are needed to satisfy the requirement of carbon sources. On the other hand, the excess lactate derived from the “Warburg effect” can also be used for ATP production and biomacromolecules synthesis [13,14]. Stromal cells can utilize lactate excreted by glycolytic tumor cells to produce pyruvate, which in turn can be extruded to refuel tumor cells. A complementary micrometabolic system is formed between anaerobic tumor cells and aerobic nontransformed stromal cells, which resembles the Cori cycle between liver and muscle [13,14]. As the nutrients is abundant for tumor cells, they will select the fastest proliferating ways rather than the maximization of ATP production. Not only glucose is vastly consumed for providing energy and carbon sources, but also glutamine is also utilized for many biosynthetic processes, such as production of nonessential amino acids, lipids, and DNA bases [5,15]. Finally, the intermediates and products of glycolysis and glutaminolysis are both used for synthesizing biomass that rapidly growing tumor cells need (Figure 2) [15]. As mentioned above, it seems increasingly clear that the metabolism of glucose to CO_2_ through mitochondrial OXPHOS to generate ATP is unfavorable for the proliferation of tumor cells.

In fact, the switch from OXPHOS to aerobic glycolysis in most tumor cells are closely associated with the mutation of genes encoding vital proteins involved in energy metabolism [2,12]. The phosphoinositide 3-kinases (PI3Ks) are a family of related intracellular signal transducer enzymes involved in the regulation of cell growth, proliferation, differentiation, survival, and intracellular trafficking. These cellular functions are associated with the capability of PI3Ks to activate protein kinase B (PKB or AKT) as in the PI3K/AKT signaling pathway. The mutation-mediated continuous activation of the PI3K/AKT signaling pathway is observed in various tumors, which can upregulate the expression of glucose transporters (GLUTs) and substantially enhance the capture of glucose into the cytoplasm by hexokinase (HK), as well as activate phosphofructokinase (PFK) or upregulate PFK expression (Figure 2) [5]. To some extent, the activation of PI3K/AKT signaling pathway results in the dependence on high level of glucose in tumor cells, which in turn facilitates aerobic glycolysis.

**Figure 3:**
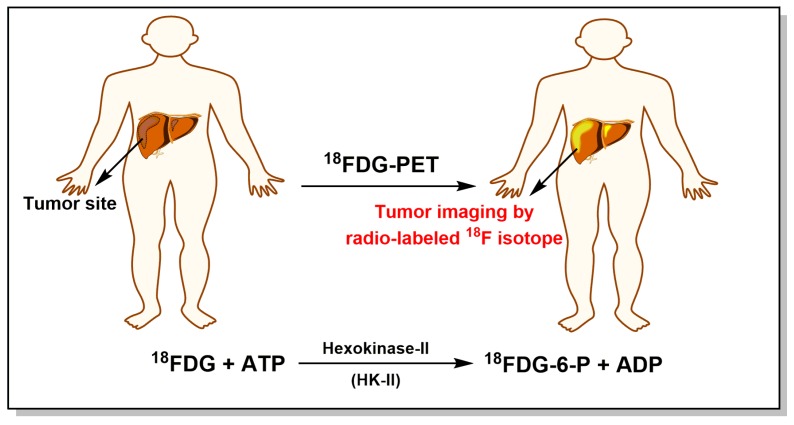
Schematic representation of tumor imaging by ^18^F-fluorodeoxyglucose positron emission tomography (^18^FDG-PET) technology.

This unique “Warburg effect” in tumor cells depends on the high rate of glucose uptake, which is accomplished by the overexpression or upregulation of GLUTs and other glycolytic enzymes [16]. It is specially noted that HK, which phosphorylates a glucose molecule to a glucose-6-phosphate (G-6-P), is responsible for the essentially irreversible first step of glycolytic process. Mammalian cells possess four HK isozymes defined as HK-I–V, in which HK-I, II, and III have a high affinity for glucose with *K*_m_ values of approximately 0.02 mM when compared to HK-IV (*K*_m_ ≈ 5 mM) [6,16,17]. HK-I–II can be regulated by feedback inhibition by their product G-6-P, while HK-IV is not allosterically inhibited by G-6-P [16]. Except for monomeric HK-IV (~50 kD), other HK isozymes are composed of two similar domains with molecular mass of approximately 100 kD [18]. The glucose and ATP binding sites of HK lie in a cleft between the two domains, once glucose binds, each domain moves toward each other to shrink the cleft [16]. The binding of glucose significantly enhances the affinity between HK and ATP molecule. In particular, HK-II is selectively expressed in tumor cells [6,13,17,19,20,21], which has been shown to be located on the outer mitochondrial membrane through specific binding to a porin-like protein, the voltage-dependent anion channel (VDAC) [22]. In this way, HK-II gets directly preferential access to utilize the new ATP generated from mitochondrial OXPHOS and protection from the inhibition of G-6-P, which leads to the conversion of glucose to G-6-P with a high rate, followed by an increased efficiency of glycolysis and biosynthesis that take place in tumors [6,15,18]. In fact, mitochondrial-bound HK-II is one of the main driving force for “Warburg effect” in tumor cells [16,18]. For example, when tumor mitochondria containing bound HK were added to liver cytosol lacking mitochondria that has a low ability to initiate glycolysis, glycolytic rate markedly increased to levels observed with tumor cytoplasm [23]. A subsequent study revealed that the isoform of mitochondria bound HK was identified as HK-II, the predominantly expressed HK isozyme in malignant tumor cells [24]. Based on this prominent phenotype of tumor cells, the “Warburg effect” has already been used for tumor imaging with ^18^F-fluorodeoxyglucose positron emission tomography (^18^FDG-PET) technology (Figure 3A) [2,5,25,26]. ^18^FDG-PET technology is based on the fact that a glucose analog (2-deoxyglucose, 2-DOG) can be phosphorylated by mitochondrial HK-II to produce 2-deoxyglucose-6-phosphate (2-DOG-6-P) that is not metabolized further and, thus, accumulates in tumor cells with much higher levels than that in normal cells, in which 2-DOG is radiolabeled with ^18^F isotope (^18^FDG). A typical hot spot on PET imaging indicates the presence of solid tumors. Now ^18^FDG-PET technology is widely used for detecting malignant solid tumors and monitoring their progress or treatment effect [25].

Glyceraldehyde-3-phosphate dehydrogenase (GAPDH), another key enzyme in glycolysis, that catalyzes the conversion of glyceraldehyde-3-phosphate (G-3-P) to 1,3-bisphosphoglycerate, with concomitant reduction of NAD^+^ to NADH [27]. In addition to be a member of glycolysis, there were several evidences that revealed GAPDH participated other nonglycolytic functions, such as cell death and neurodegenerative disorders [28,29]. Hara and coworkers used cell apoptotic stressors (staurosporine or etoposide) to activate nitric oxide synthase (NOS), leading to the *S*-nitrosylation of GAPDH at the active site Cys150 residue (Cys150 for rat and rabbit, Cys152 for human) via generated NO. *S*-nitrosylation of GAPDH augments its binding to Siah1, an E3 ubiquitin ligase, whose nuclear localization signal (NLS) mediates the nuclear translocation of the GAPDH-Siah1 protein complex. Modified GAPDH stabilizes the rapidly turning over Siah1, facilitating the degradation of its nuclear targets, which results in cell apoptosis [28]. The NO/GAPDH/Siah1 signaling cascade may also be conducive to further understand the molecular mechanism of GAPDH in neurodegenerative disorders (e.g., Alzheimer’s disease and Parkinson’s disease) [29]. Other poorly understood functions of GAPDH include cytoskeleton regulation, membrane fusion, calcium flux, DNA repair, and RNA transcription [27,29]. GAPDH is commonly overexpressed in various malignant tumors (e.g., cutaneous melanoma and colon cancer [30,31,32]), and its expression is associated with a poor prognosis [33].

Pyruvate kinase (PK), an important regulatory enzyme in glycolytic process, catalyzes the irreversible transfer of a phosphate group from phosphoenolpyruvate (PEP) to adenosine diphosphate (ADP), producing a pyruvate molecule and an ATP molecule [16]. There are four distinct isoenzymes of PK in mammals, L (liver), R (erythrocytes), M1 (muscle, heart, and brain), and M2 (in early fetal tissue and most adult tissues), which act in response to the metabolic requirements of various tissues [34]. It should be noted that M2 isoform of PK (PKM2) is usually selectively overexpressed in proliferating cells, especially in tumor cells [34]. There is both a tetrameric and dimeric form of PKM2 in terms of structure. The tetrameric form has the high binding affinity to PEP, is primarily presented on normal differential tissues and proliferating cells. On the contrary, the dimeric form has the low binding affinity to PEP and commonly exists in tumor cells [16]. The switch of tetramer to dimer facilitates the direct influx of a large proportion of glucose as carbon precursors to synthetic processes (low-activity dimer) rather than energy production (highly active tetramer). PKM2 is a phosphotyrosine (pTyr) binding protein, when pTyr binds to PKM2, the allosteric activator fructose-1,6-bisphosphate (FBP) may be released, leading to the formation of low-activity dimer and thus the inhibition of PKM2 activity [35]. When cells are stimulated by growth signaling, pTyr signaling downstream will divert glycolytic intermediates from energy production to anabolic processes through the negative regulation of PKM2. Other oncoproteins, such as Rous sarcoma virus pp60^v-src^ protein, can phosphorylate PKM2 at tyrosine residue to inhibit the enzymatic activity [36]. PKM2 activity was also reported to be modulated by the human papillomavirus type 16 E7 oncoprotein, which plays a crucial role in cell malignant transformation [37]. CARM1 (coactivator-associated arginine methyltransferase 1) is overexpressed in breast cancer to stimulate cell growth. PKM2 can be methylated by CARM1 to reversibly shift the metabolism from OXPHOS to aerobic glycolysis in breast cancer cells [38]. Thus, this regulation of PKM2 activity may underlie a foundation of the “Warburg effect”, which is favorable for the rapid growth of tumor cells. Overexpression of PKM2 has been proven to enhance the “Warburg effect” and provide a selective growth advantage for tumor cells in vivo [39].

Pyruvate dehydrogenase (PDH), a key enzyme involved in the conversion of pyruvate to acetyl coenzyme A (acetyl-CoA) and subsequent OXPHOS in mitochondria, can be negatively modulated by the upregulation of pyruvate dehydrogenase kinase (PDK) in tumor cells [15,40]. The inactivation of PDH results in a majority of pyruvate be metabolized to lactate by lactate dehydrogenase (LDH). Furthermore, LDH is also overexpressed in tumor cells, which facilitates the rate of glycolysis [16,40]. Hypoxia-inducible factor-1 alpha (HIF1α) was demonstrated to induce the overexpression of PDK and LDH [15]. Therefore, inhibition of mitochondrial PDK via pyruvate analog dichloroacetate (DCA) or RNA interference-mediated LDH inhibition may revert the “Warburg effect” to mitochondrial pyruvate metabolism in malignant tumor cells, leading to cell death [6].

For most solid tumors, hypoxia further accentuates the reliance on glycolysis, in which the GLUTs and many glycolytic enzymes would be upregulated [2,41]. However, hypoxia is not the prerequisite that tumor cells select glycolysis for cell metabolism. For instance, leukemia cells reside within the condition of higher oxygen tensions than cells in most normal tissues, but they are highly glycolytic [42]. Additionally, lung cancer cells also exhibit a high rate of glycolysis even though they are exposure to oxygen [5].

As described above, although mitochondrial function is still normal, little OXPHOS continues in tumor cells. In order to satisfy the metabolic requirements of both energy and materials for rapidly proliferating tumor cells, ~85% of the glucose is processed to lactate via glycolytic pyruvate and ~5% of the glucose is metabolized by mitochondrial OXPHOS [5] (Figure 1B). In addition, for proliferating cells, ~10% of the glucose is diverted into the upstream of pyruvate production for biosynthesis, such as the pentose phosphate pathway (PPP) [5]. This well-designed strategy provides both the energy and biomass required for infinite proliferation of tumor cells. However, the altered metabolism also becomes their Achilles heel because it provides pivotal targets for discovering newly potential antitumor drugs.

From the perspective of drug discovery and development, drugs designed to disturb the reprogrammed energy metabolism of tumor cells, especially at the glycolytic level, may exhibit potential therapeutic effects in clinical treatment of cancers [16]. In recent years, a small molecule alkylating agent, 3-bromopyruvate (3-BrPA), has attracted great attention as a promising glycolytic inhibitor because their successful preclinical trials in tumor-bearing animals [13,15,16,40,43,44].

## 3. Properties, Mechanism of Action, and Cellular Targets of 3-BrPA

**Figure 4:**
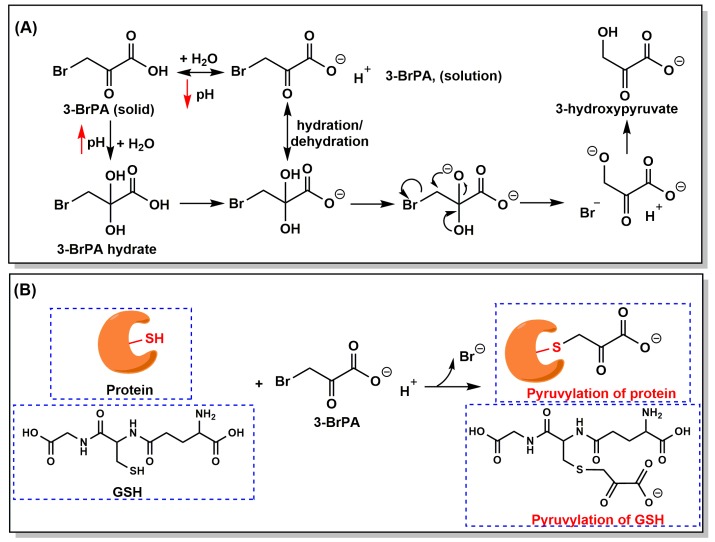
Structure, decomposition, and mechanism of action of 3-BrPA. (**A**) Proposed mechanism of decomposition in solutions with different pH values. (**B**) Modification or inactivation of proteins, enzymes, or glutathione by 3-BrPA through the covalent binding of a pyruvic moiety to the thiol groups (especially the –SH group) of targets.

3-BrPA is a halogenated analog of pyruvate (Figure 4A), which exhibits strong alkylating properties toward biomacromolecules (e.g., enzymes and proteins) since the first report in 1969 by Baker et al. [45]. Based on a chemical view, 3-BrPA is easy to attack a nucleophilic group (typically a thiol) through irreversibly covalent binding of the pyruvic moiety to target compounds, proteins, and enzymes (Figure 4B) [40]. This alkylation process occurs in a bimolecular nucleophilic substitution reaction (S_N_2) and a bromo radical is released later. The presence of an electronegative bromo group enhances the instability of the neighboring carbonyl carbon, which endows 3-BrPA with a particularly high reactivity with other nucleophiles and results in the instability of 3-BrPA in water [46]. At physiological conditions (37 °C, pH7.4), 3-BrPA was determined with a half-life of 77 min [47]. Along with the increasing pH values, the half-lives dramatically decreased. Interestingly, 3-BrPA is converted to 3-hydroxypyruvate at neutral pH and, being faster with alkaline condition, yielded HBr as a byproduct (Figure 4A) [47]. This property may favor the minimal toxicity of 3-BrPA for normal tissues, while may be one of the reasons responsible for its specific toxicity against tumor tissues because of the increased acidity of extracellular microenvironment in most solid tumors [40,47]. Thus, proper administration and precautionary solution preparation of 3-BrPA have important implications for successful treatment of tumors.

As depicted in Figure 4B, many enzymes or proteins have been reported to be covalently modified by 3-BrPA, typically at one or more cysteine (Cys) residues by directly displacing the bromide atom [13,40,43]. The pyruvylation of target proteins results in the conformational change and, subsequently, the loss of activity. In addition, modification of other amino residues within target proteins was also confirmed, such as glutamate and lysine [48,49]. The earliest report is the effect of 3-BrPA on the regulation of glutamate dehydrogenase activity that published in 1969 [45]. In 1978, Meloche et al. identified glutamate residue within the active site of 2-keto-3-deoxygluconate-6-phosphate aldolase was the alkylating site of 3-BrPA [48]. The first biochemical evidence that 3-BrPA can alkylate a Cys residue of a protein was observed in the 3-BrPA-mediated inactivation of the apodecarboxylase in 1976 [50]. Moreover, 3-BrPA was demonstrated to have the capacity to inactivate isocitrate lyase, a crucial enzyme involved in the glyoxylate cycle that only exists in plants and microbes, by the alkylation of Cys195 residue [51]. This alkylating property of 3-BrPA has made it a novel promising antifungal drug (e.g., *Mycobacterium tuberculosis*, *Cryptococcus neoformans*, and *Prototheca algae*) [52,53,54,55] and antiprotozoal drug (e.g., *Trypanosoma brucei* and *Toxoplasma gondii*) [56,57].

**Figure 5:**
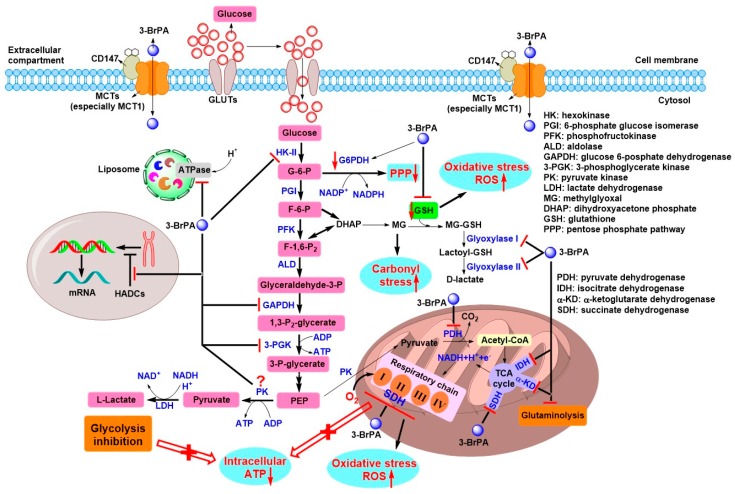
Underlying mechanism of 3-BrPA-mediated antitumor activity. 3-BrPA enters tumor cells via tumor-specific overexpression of monocarboxylate transporters (MCTs) (especially MCT1), followed by the inhibition of glycolysis (e.g., HK-II, GAPDH, and 3-PGK), mitochondrial OXPHOS (e.g., PDH, SDH, IDH, and αKD), PPP (e.g., G6PDH), glutaminolysis (e.g., IDH and αKD), the MG pathway (e.g., glyoxylase I and II), HDACs, and H^+^-vacuolar ATPase, downregulation of G6PDH and direct conjugation with GSH, leading to the decrease of intracellular ATP, an increase in oxidative stress (e.g., ROS), inhibition of anabolic process (e.g., PPP), carbonyl stress (e.g., MG), and destabilization of liposome. Consequentially, 3-BrPA selectively induces cell death by apoptosis or necrosis, while normal cell remains unaffected.

More importantly, up to date, 3-BrPA is generally considered as an efficient energy blocker, due to its ability to inhibit several key glycolytic enzymes or related metabolic enzymes, which make it a potential candidate as an antitumor drug (Figure 5) [13,15,40,43]. Since the first study on 3-BrPA’s antitumor effects were reported in 2001, HK-II is commonly considered a main target of 3-BrPA in this field [17]. The direct inhibition of mitochondrial HK-II isolated from the rabbit liver implanted VX2 tumor via 3-BrPA was demonstrated by Ko et al. [17]. In this study, a dosage of 2.4 mM and 15 mM 3-BrPA induced 50% and complete glycolytic inhibition, respectively, while 5 mM 3-BrPA completely inhibited HK-II activity; complete mitochondrial OXPHOS was observed with a much low dosage of 1.2 mM 3-BrPA [17]. 3-BrPA was believed to inhibit HK-II through a covalent modification on cysteine residues of HK-II, which directly caused its dissociation from the mitochondria with concurrent release of mitochondrial apoptosis-inducing factor (AIF) to cytosol, leading to cell death [58]. Previous studies revealed that cytochrome c release was involved in 3-BrPA-induced cell apoptosis [42], but no release of cytochrome c was found in the above study [58]. However, the following studies further showed 3-BrPA treatment resulted in an increase of cytochrome c release [59,60], along with an elevated expression of active proapoptotic caspase-3 and a decrease of antiapoptotic Bcl-2 and Mcl-1 [59]. Several recent studies indicated that 3-BrPA also inhibited the expression of HK-II at mRNA and protein levels [59,61]. Moreover, the binding of HK-II with VDAC hinders the exposure of apoptotic mitochondrial permeability pore through preventing the translocation of proapoptotic BAX protein to the outer mitochondrial membrane. Thus, the inhibition and subsequent dissociation of HK-II from mitochondria induced by 3-BrPA may trigger cell apoptosis in this way [13]. In another study performed by da Silva et al., mitochondrial HK-II in HepG2 cells was weakly affected by 3-BrPA with up to 5 mM, but low concentration of 3-BrPA induced a significant decrease in cell viability [62]. This result was also confirmed in different studies that HK-II activity remained completely unaffected by 3-BrPA at cytotoxic concentration [63,64,65]. To some extent, HK-II inhibition is only one aspect in 3-BrPA-mediated cell death, the presence of other targets may be also included.

Additionally, GAPDH was found to be inhibited by 3-BrPA in several studies performed by different research groups (Figure 5) [64,65,66]. Using a radiolabeled [^14^C]-3-BrPA, GAPDH was identified as the primary intracellular target of 3BrPA in multiple cancer cell lines via 2D gel electrophoretic autoradiography, mass spectrometry, and immunoprecipitation techniques [63]. In addition, the pyruvylation of GAPDH by 3-BrPA led to functional loss of the enzyme in cells and in vitro and in vivo [62,63,66,67,68]. In HepG2 cells, a treatment with 0.15 mM 3-BrPA for 30 min caused more than 70% inhibition of GAPDH activity [62]. In human colorectal cancer HCT116 cells, GAPDH activity was seriously inhibited by 3-BrPA with an IC_50_ value less than 30 μM [66]. A *K*i value of approximately 25 μM for this inhibition was determined in vitro [67]. Recently, Yadav et al. demonstrated that 3-BrPA treatment also decreased the expression of GAPDH at protein levels, as well as LDH and succinate dehydrogenase (SDH) [59]. Of note, GAPDH is *S*-nitrosylated by NO at the Cys150 residue, whether the Cys150 residue is at the same alkylating site of 3-BrPA? In 2017, Chen et al. revealed that 3-BrPA caused rabbit GAPDH inactivation through the pyruvylation of four cysteine residues by mass spectrometry, especially for active site Cys150 residue (equivalent to Cys152 in human) [66]. We hypothesize that, similar to NO/GAPDH/Siah1 signaling cascade, 3-BrPA-mediated pyruvylation of GAPDH may promote the nuclear translocation of this protein and induce cell death via ubiquitination and degradation of nuclear proteins.

For other enzymes involved in glycolytic process, 3-BrPA was reported to strongly inhibit the activity of 3-phosphoglycerate kinase (3-PGK) in cell extracts (Figure 5) [62]. Approximately 75% 3-PGK activity was lost in HepG2 cells upon 30 min treatment with 150 μM 3-BrPA [62]. It should be noted that this is the only one study published for investigating the inhibition of 3-PGK by 3-BrPA to now. In the same study, 3-BrPA enhanced PK activity as almost twice as that of control [62], which was further verified by Valenti and coworkers in human prostate cancer PC-3 cells [65]. Paradoxically, PK isolated from human erythrocytes was inhibited by 3-BrPA via the alkylation of an active site Cys residue, with an inactivation constant (*k*_s_) of 1.84 min^−l^ and dissociation constant (*K*_d_) of 0.14 mM [69]. Earlier, 3-BrPA was also reported to inactivate yeast PK activity by modifying the active site Cys residue [70]. However, inspiringly, a series of small molecule inhibitors of PKM2 isoform have been screened and the most potent compound induced decreased glycolysis and increased cell death [71]. This implies the need for detailed study of interaction between 3-BrPA and PK to better understand the underlying mechanism of 3-BrPA.

Recent reports showed 3-BrPA had ability to inhibit postglycolysis targets and other metabolic pathways, such as LDH, PDH, TCA cycle, and glutaminolysis (Figure 5) [72]. Dell’Antone demonstrated that 3-BrPA was a weak substrate for LDH that converted it into 3-bromolactate [67]. Later, Yadav et al. showed that 3-BrPA slightly reduced the expression of LDH [59]. However, in another two studies, LDH activity was not affected by 3-BrPA either in vitro (purified LDH) or in cell lysates [63,65]. Thus, we speculate that the formation of 3-bromolactate may not be involved in the antitumor effect of 3-BrPA, but this effect has not been validated in vivo. Although 3-BrPA was proven to be an inhibitor of PDH [72,73,74], this cannot explain the antitumor effect of 3-BrPA because it would sustain the tumor metabolic profile rather than destroying it, as noted above. Furthermore, an important discovery that 3-BrPA can inhibit SDH, the complex II of mitochondrial respiratory chain, also the part of TCA cycle, was first reported by Sanborn et al. in 1971 [75]. This effect was further confirmed in several studies published in recent years [59,62,65,72]. By the impairment of SDH activity, 3-BrPA strongly inhibited substrate succinate and ADP-driven mitochondrial respiratory in HepG2 cells with an IC_50_ value of 150 μM. Interestingly, at the IC_50_ level of SDH inhibition (~20 μM), no obvious inhibition of mitochondrial respiration was found; however, a significant decrease of respiratory rate was observed with more than this inhibitive level [62]. In fact, in the first antitumor study by Ko et al., 3-BrPA also exhibited complete inhibition of mitochondrial respiration with a higher dosage of 1.2 mM [17]. These results reflect that SDH (complex II) inhibition partly influences the respiration, because other targets (e.g., complex I, III) in respiration chain are also involved [62,65]. A comprehensive study performed by Jardim-Messeder et al. showed that 3-BrPA significantly inhibited TCA cycle and glutaminolysis through inhibition of isocitrate dehydrogenase (IDH), α-ketoglutarate dehydrogenase (αKD), and SDH at low micromole concentration [72]. Although glutaminolytic enzymes, such as glutaminase (GLS) and glutamate dehydrogenase (GDH), were not directly affected by 3-BrPA, this inhibition of TCA cycle can lead to the impairment of glutaminolysis due to α-KG generated from glutamine is incorporated into the TCA cycle by IDH and αKD activities (Figure 2 and Figure 5) [72]. The impaired glutaminolysis may affect the supplement of building blocks for biomass synthesis. In this study, 3-BrPA also inhibited mitochondrial respiration chain complex I and complex II, but not IV. Additionally, 3-BrPA distinctly decreased the reduced thiol groups in HepG2 cells, indicating that the reduced thiol groups can be the target of 3-BrPA [72]. Indeed, a remarkable decrease of reduced glutathione (GSH) level was observed after 3-BrPA treatment in both microorganisms and various tumor cells [53,61,65]. There are two possible explanations responsible for this phenomenon: (1) GSH scavenges reactive oxygen species (ROS) induced by 3-BrPA [65,76,77] and (2) 3-BrPA directly reacts with GSH forming an *S*-conjugate (Figure 4B) [66,78,79]. Expression of genes encoding the enzymes (e.g., γ-glutamylcysteine synthetase) involved in GSH metabolism was also affected by 3-BrPA [53,76,80]. In addition to the above targets mentioned, as shown in Figure 5, 3-BrPA was reported to inhibit other metabolic targets, such as glyoxylase I and II, the two enzymes responsible for the methylglyoxal (MG) pathway that scavenges toxic MG to produce D-lactate [65]; histone deacetylase (HDAC) I and III, enzymes responsible for epigenetic modification [81]; and H^+^-vacuolar ATPase, which produces acidic compartments (e.g., lysosomes) [82].

## 4. Antitumor Effects of 3-BrPA and the Underlying Mechanism

At first sight, it seems unlikely to consider 3-BrPA as a candidate antitumor drug as it is highly reactive electrophilic agent with nonspecific reaction with many biomacromolecules. As stated, most known targeted enzymes or proteins of 3-BrPA are involved in tumor energy metabolism, especially for the glycolytic pathway, as well as the TCA cycle and OXPHOS to some extent. Thus, 3-BrPA-mediated inhibition of ATP production and anabolic metabolism can be utilized as a powerful tool for tumor treatment. From the view of drug discovery, based on the current understanding of 3-BrPA, the potential of 3-BrPA for tumor treatment is worth a try.

### 4.1. Initial Antitumor Studies and Cell Death Induced by 3-BrPA

Fortunately, the first antitumor study of 3-BrPA was reported in 2001 by Pedersen’s research group at Johns Hopkins University [17]. In this study, they found that 3-BrPA successfully killed AS-30D hepatocellular carcinoma (HCC) cells via the inhibition of both ATP-producing glycolysis and mitochondrial respiration [17]. One year later, they demonstrated that intraarterial delivery of 3-BrPA into liver-implanted rabbit VX2 tumors selectively caused the death of most cells within tumor while surrounding liver tissue remained unaffected [83]. Even systemic delivery of 3-BrPA also did not caused obvious harm to normal organs [83]. Their further study showed that 3-BrPA selectively induced ATP depletion and cell death in HCC cells rather than normal hepatocytes [84]. Importantly, advanced AS-30D hepatic tumors were completely eradicated in all treated rats by 3-BrPA without apparent systemic toxicity or recurrence, as also indicated by PET imaging [84]. In another in vivo mouse model of hepatoma, mean tumor volume and tumor growth rate were significantly reduced in 3-BrPA-treated mice in a dose-dependent manner when compared to the control mice [19]. This study also showed that 3-BrPA induced apoptosis of mouse HCC cells in vivo by inhibiting HK-II, facilitating the its dissociation from VDAC to release apoptotic cytochrome c and thus activate mitochondrial apoptotic signals [19]. The mitochondrial apoptotic effect derived from HK-II inhibition was also reported by other groups [41,58]. In 3-BrPA-mediated apoptotic effect, dephosphorylation of proapoptotic protein BAD at the Ser112 residue allowed the translocation of BAX to mitochondria, followed by a change in membrane permeability and the release of cytochrome c and AIF [42,58]. In this case, proapoptotic caspase 3 activation was usually observed and presented as cleaved form [41,42,59,63,68,85,86,87]. Moreover, 3-BrPA also affected the levels of poly(ADP-ribose) polymerase (PARP), cleaved PARP, antiapoptotic Bcl-2, and Mcl-1 [59,80,85,87,88]. In fact, 3-BrPA treatment led to both apoptotic and necrotic cell death. Because of apoptosis is ATP-dependent process, when low dosage of 3-BrPA was used, ATP depletion was moderate; this situation mainly led to apoptosis because remained cellular ATP was sufficient to execute apoptosis. On the contrary, with high dosage of 3-BrPA, ATP was completely depleted and only necrosis occurred [42,86]. For example, in HL60 human myeloid leukemia cells, 3-BrPA induced both apoptosis and necrosis at 20–30 μM and an almost exclusively necrotic response at 60 μM [86]. The special manner of cell death induced by 3-BrPA has been supported by multiple in vitro and in vivo studies [19,41,53,60,77,88,89].

### 4.2. Role of GSH and ROS on the Antitumor Effects of 3-BrPA

It is well known that the PPP pathway provides carbon sources for nucleotide synthesis, as well as produces NADPH as a source of cellular reducing equivalents to maintain the intracellular redox state, especially for generating GSH, which is a major protective factor for cells from the damage derived from oxidative stress (e.g., ROS) [5]. Since the G-6-P is the first substrate of PPP pathway, therefore, 3-BrPA-mediated inhibition of HK-II may block not only the glycolytic pathway, but also the anabolic process and the production of reducing equivalents, leading to the occurrence of more cytotoxic oxidative conditions [13]. Furthermore, G-6-P dehydrogenase (G6PDH), which converts G-6-P to 6-phosphoglucono-δ-lactone (the first step of the PPP (Figure 2)), was also reported to be downregulated by 3-BrPA (Figure 5) [90]. In addition, ROS can be produced by the impairment of SDH/complex II (Figure 5) [91], and 3-BrPA could inhibit this respiratory enzyme. Indeed, the increase of ROS and concomitant decrease of GSH were commonly found in 3-BrPA-mediated antitumor studies [53,59,61,64,65,76,77,86,89]. To justify the role of ROS in the antitumor effect of 3-BrPA, ROS scavenger N-acetylcysteine (NAC) completely blocked the cell killing triggered by 3-BrPA in different tumor cell lines [65,77,89]. Interestingly, higher levels of GSH were observed in 3-BrPA-resistant melanoma cell lines when compared to sensitive cell lines [77]. After exposure to 200 μM 3-BrPA, sensitive melanoma cells exhibited GSH reduction and 2–2.5-fold increase of ROS level. A complete protection towards 3-BrPA-induced cell death was shown by NAC or GSH addition in sensitive cells. In particular, when *S*,*R*-sulfoximine (BSO, an inhibitor of glutathione synthesis) was added, GSH levels were dramatically decreased in resistant melanoma cells and they again became sensitive to killing by 3-BrPA [77]. Calviño et al. further demonstrated that intracellular GSH was a crucial determinant of 3-BrPA toxicity and GSH depletion-dependent p38 mitogen-activated protein kinase (MAPK) activation enhanced 3-BrPA toxicity, which was compromised by extracellular signal-regulated kinase (ERK) and AKT activation [86]. For this study, phosphorylation-activation of AMP-activated protein kinase (p-AMPK), which was negatively regulated by AKT activation, also contributed to 3-BrPA toxicity [86]. In a recent study, a combination of 3-BrPA with BSO effectively suppressed cell viabilities of anoikis-resistant (AR) HCC cells through apoptosis by inhibiting glycolysis and increasing ROS levels [80]. Meanwhile, 3-BrPA plus BSO effectively suppressed tumor growth when compared to other groups treated with 3-BrPA or sorafenib alone in mice tumor xenograft AR HCC models [80]. Moreover, the synthesis of GSH is an ATP-consuming process and, therefore, 3-BrPA-induced ATP depletion would shift cells to oxidative status. More importantly, the direct conjugation between 3-BrPA and GSH further facilitates the generation of ROS. These results altogether indicate that GSH depletion along with ROS generation are pivotal for the antitumor effects triggered by 3-BrPA.

### 4.3. Specific Tumor Selectivity of 3-BrPA

As previously mentioned, 3-BrPA displayed excellent antitumor effects without obvious side effects in initial antitumor studies in vivo. The current available explanations for tumor specificity of 3-BrPA are attributed to three aspects [40]: (1) specific overexpression of mitochondria-bound HK-II and high rate of glycolysis in tumor cells; (2) tumor cells selectively overexpress monocarboxylate transporters (MCTs), a family of transmembrane transporters, which are known to export the excess lactate excreted by tumor cells to avoid the intracellular acidification and cell death; and (3) acidic extracellular microenvironment of tumor tissues.

As an effective inhibitor of glycolytic enzymes, especially for HK-II and GAPDH overexpressed in most tumor cells, in this case, we can keep in mind that 3-BrPA can selectively target tumor cells characterized by a high glycolytic phenotype.

On the basis of the “Warburg effect”, tumor cells have increased uptake of glucose associated with lactate production. To extrude the excess lactate produced by glycolysis, tumor cells commonly overexpress MCTs, especially MCT1 and MCT4, to transport the excess lactate to extracellular environment via a proton-linked mechanism, leading to an acidic extracellular microenvironment that promotes invasion and metastasis [92]. In addition, lactate produced by LDH can be again taken up by aerobic tumor cells via MCTs to resupply cell growth. Given the structural similarity of 3-BrPA with lactate, 3-BrPA may enter tumor cells via the same lactate transporter MCTs. This hypothesis has been confirmed in several studies. Initially, sodium-coupled monocarboxylate transporter 1 (SMCT1)—a sodium (Na^+^)-dependent electrogenic transporter of short-chain fatty acids (e.g., acetate, propionate, and butyrate), B-complex vitamin nicotinic acid, and monocarboxylates (e.g., lactate and pyruvate)—was found to be a transporter of 3-BrPA [81]. In MCF-7 cells transfected with *SLC5A8* gene that coding SMCT1, 3-BrPA induced significant apoptosis when compared to vector-transfected cells, in which this apoptotic effect was associated with the inhibition of histone deacetylase 1 (HDAC1) and HDAC3 mediated by 3-BrPA [81]. However, as a tumor suppressor, SMCT1 was epigenetically downregulated in a variety of tumors through DNA methylation during carcinogenesis [13,93,94]. Therefore, it is speculated that the uptake of 3-BrPA into cells may be mediated by other membrane transporters rather than SMCT1. It must be mentioned that a study published in Nature Genetics by Birsoy and coworkers in 2013, they performed a genome-wide haploid genetic screen to identify the *SLC16A1* gene product MCT1, which was found to be the main determinant of 3-BrPA sensitivity (Figure 5) [95]. In this study, compared to wild-type KBM7 cells expressing MCT1, MCT1-null cells were resistant to the toxicity and metabolic effects of 3-BrPA and did not take up [^14^C]-labeled 3-BrPA, which indicated that 3-BrPA might not enter cells in the absence of MCT1 and clearly showed that MCT1 as a primary transporter of 3-BrPA [95]. In accordance with the pH dependence of MCT1-mediated transport [92], an increase in extracellular acidity promoted cellular uptake of 3-BrPA [95]. Indeed, the decrease of the extracellular pH from 7.4 to 6.0 resulted in a reduction of the IC_50_ values for 3-BrPA cytotoxicity in three breast cancer cell lines [96]. The uptake and cytotoxicity of 3-BrPA were strongly decreased by MCTs inhibitors, especially the MCT1 inhibitor, suggesting that MCT1 plays a key role in 3-BrPA uptake thereby affecting its cytotoxicity. Furthermore, the hyperglycosylation of chaperonin CD147 is a prerequisite for MCT1 activity (Figure 5), in which inhibition of CD147 glycosylation by tunicamycin decreased the expression of MCT1, leading to a reduction in 3-BrPA uptake [96].

As described previously, the efflux of lactate via MCTs produces an acidic extracellular milieu of tumors that contributes to 3-BrPA stability [47]. At 37 °C, in 0.10 M K_3_PO_4_ buffer, 3-BrPA decay half-lives were found to be 430, 160, 77, and 37 min at pH 6.5, 7.0, 7.4, and 8.0, respectively. It was obvious that at pH of 6.5–7.0—a typical extracellular acidity of most tumors—the half-lives of 3-BrPA were notably longer, while at physiological condition of normal tissues (37 °C, pH 7.4), a significantly short half-life (77 min) was determined [47]. In addition, at acidic extracellular conditions, the affinity for 3-BrPA uptake via MCTs in different tumor cells was higher than that at physiological conditions [96]. These would favor the special toxicity of 3-BrPA for acidic tumor tissues while normal tissues remain minimal toxicity or unharmed.

### 4.4. Chemosensitivity of 3-BrPA with Other Antitumor Drugs In Vitro and In Vivo

Considering that the complex process of cancer biology, multiple proteins, enzymes, signaling pathways, or other biological mechanisms are involved to bypass the therapeutic effects mediated by antitumor drugs [2,97,98]. It is extremely challenging to discovery a single monofunctional drug with desirable therapeutic effects for most refractory cancers. Combination treatment, in which two or more drugs that act by different mechanisms are used simultaneously in a prescribed therapeutic regimen, may be a promising therapeutic strategy to effectively kill tumor cells and reduce the possible occurrence of resistance [99,100]. On the basis of tumor specificity and multiple inhibition in cellular targets of 3-BrPA, it may be possible to reduce the tumor resistance when 3-BrPA is administrated with other chemotherapeutic drugs. Meanwhile, the dosages of chemotherapeutic drugs can be largely reduced, thereby avoiding or maximally decreasing the adverse effects.

In fact, HL60/AR cells that express a multidrug resistance (MDR) phenotype for the antitumor agents doxorubicin (DOX), vincristine, and ara-C, remained sensitive to 3-BrPA alone, which resulted in a similar sensitivity in both MDR HL60/AR and parental HL60 cells [42]. At dosage of 50 μM 3-BrPA, which alone only produced less than 10% apoptosis within 24h, essentially enhanced the killing effects of DOX, vincristine, or ara-C in resistant HL-60/AR cells [42]. Of note, a recent study indicated that simultaneous inhibition of glycolysis and abrogation of *c-myc* expression by 3-BrPA and bromodomain-containing protein 4 (BRD4) inhibitor ITH-47 that prevents the transcription of *c-myc* gene, a synergistic cell killing effect was obtained on U937 myeloid leukemia cells [101]. However, in this study, cell death occurred primarily through apoptosis and not necrosis.

In two pancreatic cancer cell lines, 3-BrPA preferentially inhibited glycolysis and sensitized cells to the killing effects of heat-shock protein 90 (HSP90) inhibitor geldanamycin by 17- to 400-fold through the increased degradation of HSP90 client protein [102]. Combination of a very small dose of both 3-BrPA and geldanamycin exhibited synergistic in vivo antitumor effects in mice xenograft models of pancreatic cancer by >75% inhibition of tumor growth, significantly increasing the median survival rate, while no any single treatment had effects on tumor growth or median survival rate compared with the control groups [102]. When 3-BrPA was combined with cisplatin or oxaliplatin with non-toxic low-dose, 3-BrPA strikingly enhanced the antiproliferative effects of both platinum drugs in HCT116 cells and resistant p53-deficient HCT116 cells [89]. The authors proposed the potential of 3-BrPA as chemosensitizer was due to effects not only on glycolysis, but also on mitochondria and ROS production, in which ATP deprivation may help overcome resistance through decreasing macromolecule synthesis and DNA repair [89]. This was corroborated in another study that intracellular ATP levels played a central role in regulating chemoresistance in human colon cancer cells [103]. Direct delivery of ATP packaged in liposomal vehicles was sufficient to render drug-sensitive HT29 and HCT116 cells become resistant. Reversely, ATP depletion induced by a moderate dose of 3-BrPA resensitized both cross-resistant HT29-OxR and HCT116-OxR cells to multiple chemotherapeutic drugs, e.g., oxaliplatin and 5-fluorouracil (5-FU) [103]. 5-FU is the first-line drug for human colorectal cancer, however, short half-life and development of resistance often render it to be administrated at large doses with high frequencies, leading to multiple side effects [104]. Recently, Chong et al. demonstrated that 3-BrPA plus 5-FU showed synergistic antitumor effects against human colorectal cancer both in vitro and in vivo by induction of mitochondria-dependent apoptosis, in which proapoptotic BAX increased and antiapoptotic Bcl-2 decreased [105]. Moreover, an increase of ROS and upregulation of p53 and p21 were observed. Importantly, combined treatment significantly suppressed tumor growth in the BALB/c mice, whereas the hepatotoxicity and nephrotoxicity were minimal [105]. Using 3-BrPA as a sensitizer to target glycolysis of breast cancer cells, the antitumor effects of tamoxifen were markedly improved through multiple modulation in apoptosis, angiogenesis, oxidative stress, and metastatic potential in vitro and in vivo [61]. In addition, 3-BrPA sensitized human breast cancer cells to tumor necrosis factor-related apoptosis-inducing ligand (TRAIL) via the p-AMPK-mediated upregulation of death receptor 5 (DR5) [106], similar to the mechanism described in a previous study [86]. In mice tumor xenograft of MCF-7 cells, drug treatments resulted in a typical necrotic area within tumors, a synergistic antitumor effect of 3-BrPA and TRAIL was observed without evident hepatotoxicity and nephrotoxicity as well [106].

Generally, most tumor cells exhibit the MDR phenotype via enhancing the expression of ATP-binding cassette (ABC) transporters, including ABCB1 (MDR1, P-glycoprotein), breast cancer related protein (BCRP, ABCG2), and multidrug resistance-associated protein 1 (MRP1, ABCC1), which are located in the cell membrane and highly dependent on ATP for activity as efflux pumps [107]. Thus, inhibition of ATP production by 3-BrPA may overcome the resistance mediated by ATP-dependent MDR, resulting in increased intracellular levels of chemotherapeutic agents. As demonstrated by Nakano et al., 3-BrPA-mediated glycolytic inhibition preferentially reduced the ATP production and induced cell death in malignant tumor cells, and enhanced accumulation and retention of daunorubicin (DNR) and mitoxantrone (MIT) in ABC transporter-expressing cells [108]. Interestingly, 3BrPA also effectively impaired ATP production and drug efflux function in side population (SP) cells, which display cancer stem cell-like characteristics and appear to be involved in chemoresistance and tumor relapse [108]. Moreover, 3-BrPA substantially increased the cytotoxic effects of DNR and DOX, even at low dosages, in human leukemic KG-1 or myeloma RPMI8226 cells, and significantly delayed tumor growth in mice implanted subcutaneously with RPMI8226 cells when combined with DOX, while no obvious side effects were observed [108]. In DOX-resistant human neuroblastoma cells, 3-BrPA was proved to overcome the DOX resistance both in normoxic and hypoxic conditions [109]. In MCT1-positive MCF-7 cells, 3-BrPA also enhanced DNR accumulation and DNR-induced cytotoxicity through reducing ATP level to inactivate MDR mechanism, but not in MCT1-negative MDA-MB-231 cells [110]. Cotreatment of 3-BrPA plus DNR markedly suppressed subcutaneous tumor growth in vivo in nude mice implanted with MCF-7 cells, and promoted DNR accumulation in tumors [110]. Similar chemosensitivity was showed by Wu et al. that 3-BrPA reversed the P-glycoprotein-mediated MDR in drug-resistant MCF-7/ADR variant cells after exposure to DOX, paclitaxel (PTX), DNR, and epirubicin (EPI) in vitro, and to EPI in mice xenograft models in vivo, where the toxicity of this regimen was tolerable as well [111]. Interestingly, in a recent study, 3-BrPA exhibited an inhibition of P-glycoprotein expression, which in turn sensitized Dalton’s lymphoma (DL) and MCF-7 cells to the cytotoxic effects of cisplatin (5 μM) even at a very low concentration of 1 μM [59].

Another viewpoint indicated that the overexpression of ABC transporters (e.g., ABCB1, BCRP, and MRP2) contributed to a large amount of intracellular ATP consumption, which in turn increased the antitumor effects of 3-BrPA [112,113]. Indeed, valproate sensitized human glioblastoma T98G cells to cell death induced by 3-BrPA via the increased expression of MRP2 or BCRP that resulted in lower ATP levels compared to 3-BrPA alone treatment group [113].

### 4.5. Clinical Studies of 3-BrPA for Tumor Treatment

As demonstrated in recent in vivo antitumor studies, few studies reported serious adverse effects induced by 3-BrPA [87] or combination of 3-BrPA and other chemotherapeutic agents [80] at their respective experimental conditions. In fact, the toxic side effect of 3-BrPA was dose-dependent, low dosage of 3-BrPA (e.g., 1.75 mM) was effective to induce death of tumor cells in vitro and in tumor-bearing animal models without obvious systemic toxicity [114,115], whereas a high dosage of 3-BrPA (e.g., 25 mM) caused considerable toxicities in both the liver and gastrointestinal system [116]. To our knowledge, for combination treatment, only one study reported the evident side effects of 3-BrPA plus BSO in mice tumor xenograft AR HCC models, although tumor growth rates in combination treatment group were significantly lower than those in the control, sorafenib, or 3-BrPA alone treatment groups [80]. Except for reasons responsible for the tumor specificity of 3-BrPA described above, another proposed explanation may be due to the selective interaction of 3-BrPA with serum proteins could contribute to the apparent loss of tissue toxicity [117].

To date, no available clinical trials regarding 3-BrPA approved can be found in website of https://www.clinicaltrials.gov/, which is a database of privately and publicly funded clinical studies conducted around the world. However, to the best of our knowledge there are still two case studies that reported the treatment of voluntary patients having advanced tumors with 3-BrPA [118,119]. In 2012, Ko et al. reported the use of a specially formulated 3-BrPA for treating a 16-year-old boy with advanced fibrolamellar hepatocellular carcinoma using transcatheter arterial chemoembolization delivery method [118]. In fact, there were almost no other available therapeutic methods can be adopted for treating the patient at that time. During several months’ treatment, no major cytotoxicity was observed for the patient, who survived a much longer period with a higher quality of life than expected. Finally, two years after the first diagnosis, the patient died due to an overload of liver function rather than the tumor itself [118]. In another case study reported in 2014 by El Sayed et al., a 28-year-old man with stage IV metastatic melanoma received the treatment of 3-BrPA through slow intravenous infusion (1–2.2 mg/kg) [119]. The tumor progression was monitored by serum LDH level, which is a highly predictive marker of tumor activity in clinical trials [120,121]. The serum LDH level moderately decreased one day after the first infusion of 3-BrPA, however, it again started to rise and reached a peak value four days later. After that, the patients received continuous six doses of 3-BrPA over the next 10 days, the serum LDH level still did not drop to normal range although it was the half of the initial high level. Considering the fact that high cellular GSH content commonly makes melanoma cells resistant to 3-BrPA, the patient received a concurrent administration of GSH scavenger (paracetamol) and 3-BrPA, followed by a sharp decline of serum LDH level. In this case, unformulated (free) 3-BrPA administration was observed with minimal hepatic, renal, and hematologic toxicity except burning sensation at the infusion site. Unfortunately, the patient died because of respiratory distress and hypoxemia [119].

It should be noted that two recent studies comprehensively described the effects of 3-BrPA on the homeostasis and tumor microenvironment (TME) of tumor-bearing animals, which would assist in optimizing therapeutic regimen of 3-BrPA in future clinical oncology [60,122]. In the first study, 3-BrPA exhibited protective and recuperative effects on the immunological, splenic, hepatic, and renal homeostasis in DL tumor-bearing mice, including reversion of tumor growth associated thymic atrophy and organomegaly (splenomegaly, hepatomegaly, and renomegaly), activation of tumor associated macrophages mediated by IFN-γ, increase of blood leukocytes (CD4+, CD8+ T lymphocytes, and NK cells) and IL-2 receptor expressing cells, alteration of serum cytokine balance, reduction of aspartate aminotransferase (AST), alanine aminotransferase (ALT), and alkaline phosphatase (ALP) [122]. In another study carried out by the same research group, they further investigated the effect of 3-BrPA on tumor growth regulatory components of TME in vivo [60]. A series of soluble and biophysical components of TME (e.g., NO, glucose, lactate, ROS, IFN-γ, IL-2, VEGF, macrophages, NK cells, CD4+, and CD8+ cells), key regulatory proteins involved in glucose uptake, intracellular pH and cell survival (e.g., GLUT1, MCT1, V-ATPase, HSP70, fatty acid synthase (FASN), and cytokine signaling-5 (SOCS-5)) and key functional markers (e.g., CD25 (IL-2 receptor), CD62L (L-selectin), TLR-4, and CD11c) were significantly altered with 3-BrPA treatment [60]. All these alterations related to TME would contribute to decrease the systemic toxicity and induce tumor regression after exposure to 3-BrPA.

## 5. Novel Chemotherapeutic Strategies of 3-BrPA

Although the amazing results obtained from in vitro cell culture and tumor xenograft animal models, some practical problems and obstacles may occur during the clinical application of 3-BrPA, especially for unformulated 3-BrPA [123]. For example, 3-BrPA induced burning sensation at vein during intravenous infusion [119] and rapid inactivation by the conjugation of GSH and serum proteins at thiol groups [78,117]. Notably, it is very likely that the small molecular size of 3-BrPA hinders its retention in tumor tissues due to the enhanced permeability and retention (EPR) effect [123]. In addition, 3-BrPA could not cross the blood–brain barrier (BBB) [124], thereby limiting its use for nervous system tumors. Importantly, in 2016, three patients died within a few days after receiving 3-BrPA through a nonmedical practitioner in Germany, and the prosecutor had begun to investigate whether improper use of 3-BrPA may constitute this involuntary manslaughter [125]. Thus, the development of novel chemotherapeutic strategies using 3-BrPA for cancer treatment is warranted.

**Figure 6:**
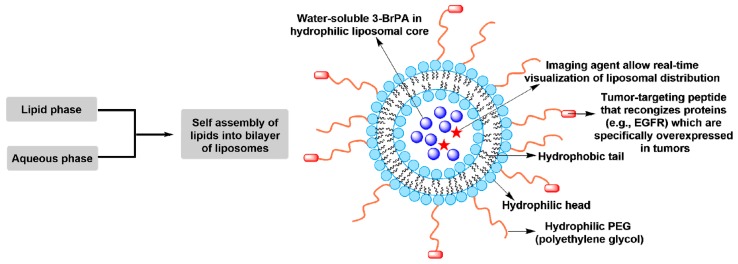
Stable liposomal nanoparticle formulation for selectively delivering 3-BrPA to tumors.

According to the suggestions proposed by mainstream scientists [118,123], unformulated 3-BrPA should not be used in clinical oncology. Alternatively, 3-BrPA had been better formulated with β-cyclodextrin (β-CD) [126], aerosol [87,127], biodegradable polymer wafer [124], propyl ester [128], perillyl alcohol [66], and liposome nanoparticle [129,130] that markedly suppressed tumor cell growth in vitro and in vivo animal models. Indeed, systemic delivery of 3-BrPA, microencapsulated into a complex with β-CD (β-CD-3-BrPA), into an orthotopic mouse xenograft tumor model of pancreatic cancer exhibited minimal or no tumor progression, while gemcitabine or β-CD alone treatment resulted in 60-fold and 140-fold increase of tumor activity over baseline, respectively [126]. No organ toxicity or tissue damages were observed in tumor-bearing mice treated with β-CD-3-BrPA compared to that treated with unformulated 3-BrPA [126]. Ming Zhou’s group showed that aerosolized 3-BrPA alone or in combination with rapamycin significantly inhibited lung tumorigenesis without any liver toxicity in mouse lung tumor models [87,127]. Utilizing a biodegradable polymer wafer formulation of 3-BrPA by intracranial delivery significantly increased survival in an animal model where a high-grade glioma was obtained, meanwhile displaying no neurological or systemic toxicity [124]. 3-Bromo-2-oxopropionate-1-propyl ester (3-BrOP), a stable ester derivative of 3-BrPA that is hydrolyzed by cellular esterase to generate 3-BrPA, exhibited more potent antitumor activities in vitro and in vivo [31,128]. As we know, the cytotoxicity of 3-BrPA is dependent on the transport mediated by MCT1 that limits its efficacy against only MCT1-expressing cells [95]. In 2017, Chen et al. reported a MCT1-independent perillyl alcohol-conjugated analog of 3-BrPA, which induced obvious cytotoxicity in highly 3-BrPA-resistant tumor cells with negative MCT1 [66]. In view of the advantages of nanotechnology-based drug delivery, several studies employed the stable liposomal nanoparticles for selectively delivering 3-BrPA to tumors (Figure 6) [129,130]. To further improve the tumor targeting, a specific peptide targeting tumor-specific receptors (EGFR) or proteins (fibrin–fibronectin complexes) was usually linked to the surface of nanoparticles. Indeed, these liposomal formulations of 3-BrPA selectively targeted tumors in 3D-spheroid and tumor-bearing mice models compared to unformulated 3-BrPA [129,130]. In in vivo animal models the 3-BrPA nanoparticle markedly suppressed tumor growth after intravenous infusion and no detectable severe adverse effects, especially the hepatoxicity, were observed [130].

Overall, these novel chemotherapeutic strategies may effectively and safely deliver 3-BrPA to tumor sites in vivo, represent a major improvement of current unformulated glycolytic inhibitors.

## 6. Conclusions and Future Perspective

Tumor cells highly depend on aerobic glycolysis (Warburg effect) to produce energy even in the presence of oxygen. Although aerobic glycolysis is ineffective in terms of ATP generation compared to OXPHOS, it confers tumor cells with a proliferating advantage because it can facilitate the uptake of glucose and provide glycolytic intermediates to synthesize the biomass needed to produce new daughter cells. In accordance with this metabolic alteration, tumor-specific overexpression and modulation of GLUTs and specific glycolytic enzymes are commonly found in various tumor cell lines, such as HK-II, PFK, GAPDH, PKM2, PDH, LDH, and so on, which in turn underlies the basis of aerobic glycolysis. Thus, tumor-specific aerobic glycolysis may be potential therapeutic target for tumor therapy. As a small molecule alkylating agent, 3-BrPA could rapidly inactivate many cellular targets via the covalent modification of thiol groups, particularly for the sulfydryl in Cys residue of target protein. Currently, it is generally accepted that 3-BrPA exhibits antitumor activity through the combined inhibition on glycolysis/mitochondrial OXPHOS-dependent ATP production, ATP-dependent MDR, tumor angiogenesis, invasion, metastasis, the induction of oxidative stress (ROS), and the regulation of pro-/antiapoptotic kinase signaling pathways. Importantly, 3-BrPA may specifically enter tumor cells via lactate transporter MCT1 that is overexpressed in most tumor cells. Furthermore, the acidic extracellular microenvironment of tumor favors the stabilization and cellular uptake of 3-BrPA. Indeed, 3-BrPA alone or in combination with other antitumor drugs exhibited significant cytotoxicity in multiple tumor cells in vitro and markedly suppressed tumor growth in animal models in vivo. Notably, no serious systemic toxicity and organ damages were observed in tumor-bearing animals in most situations, only few studies reported that 3-BrPA administration produced obvious adverse effects (especially the hepatotoxicity).

However, in clinical cases, intravenous infusion of unformulated 3-BrPA produced burning venous sensation, and high GSH level and serum proteins may also inactivate 3-BrPA. The relatively long distance from administration site to the tumor sites also renders the inactivation of 3-BrPA easily. EPR effect may also hinder the retention of 3-BrPA with small molecular size. In addition, 3-BrPA only kills MCT1-expressing tumor cells. The mainstream scientists said that unformulated 3-BrPA may be harmful in most cases in clinical setting. This implies an urgent need of novel drug delivering strategies for 3-BrPA in future, among which, wafer, liposomal nanoparticle, aerosol, and conjugate formulations have achieved better tumor-specific delivery and exhibited excellent antitumor effects in animal models. More importantly, no detectable obvious side effects were observed. We consider that these novel chemotherapeutic strategies of 3-BrPA would provide an effective tool for the application of 3-BrPA, as well as other glycolysis-based drugs in future clinical oncology. Finally, the intelligent use of 3-BrPA and related drugs needs a further elucidation of tumor metabolism and identification of the responsive signaling pathways that presenting the vulnerability for cell survival.
